# Supplementary material for: Identification of photoperiod-induced specific miRNAs in the adrenal glands of Sunite sheep (Ovis aries)
Source: Front Vet Sci. 2022 Jul 22;9:888207. doi: 10.3389/fvets.2022.888207 (PMC9354845; doi:10.3389/fvets.2022.888207)
Supplement: Supplementary file 1 [file Data_Sheet_1.ZIP › Supplementary file/miRDeep2 file.zip!/miRDeep2 file/Know_Structure/SP42A1/oar-mir-544.pdf]

miRBase precursor : oar-mir-544
Total read count : 382
oar-miR-544-5p read count : 359
oar-miR-544-3p read count : 18
remaining reads : 5

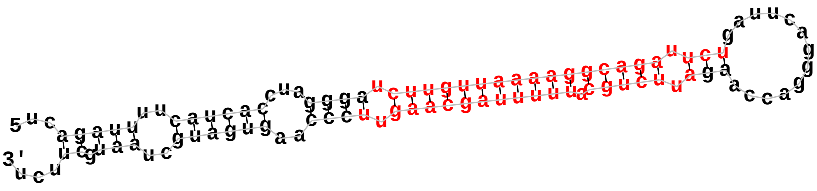

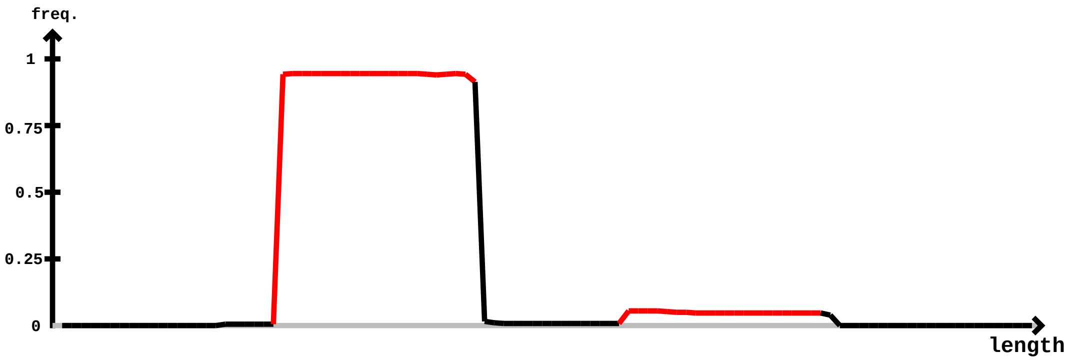


```
                                           oar-miR-544-5p
                                                                                 oar-miR-544-3p
5'- ucagauuuucaucaccuagggaucuuguuaaaaggcagauucugauucagggaccaagauucugcauuuuuagcaaguucccaagugaugcuaaugcuucu   -3'   exp
    ..(((((..((((((...((((.((((((((((((((((.(((.............))).))))).))))))))))).))))..))))))..))).))...    reads    mm    sample
    ................uagggaucuuguuaaaaggca................................................................    1        0     seq
    ................uagggaucuuguuaaaaggcag...............................................................    1        0     seq
    ......................ucuuguuaaaaggcagauu............................................................    2        0     seq
    ......................ucuuguuaaaaggcagauuc...........................................................    11       0     seq
    ......................ucuuguGaaaaggcagauucu..........................................................    2        1     seq
    ......................ucuuCuuaaaaggcagauucu..........................................................    1        1     seq
    ......................ucuuguuaaaaggcagauucu..........................................................    307      0     seq
    ......................ucuuguuaaaaggcagaCucu..........................................................    1        1     seq
    ......................Ccuuguuaaaaggcagauucu..........................................................    3        1     seq
    ......................ucuuguuaaaaggcagauucA..........................................................    24       1     seq
    ......................ucuuguuaGaaggcagauucu..........................................................    1        1     seq
    ......................ucuuAuuaaaaggcagauucu..........................................................    1        1     seq
    ......................ucuuguuaaaaUgcagauucu..........................................................    1        1     seq
    ......................ucuuguuaaaaggUagauucu..........................................................    1        1     seq
    ......................ucuuguuaaaaggcagauucuA.........................................................    2        1     seq
    ......................ucuuguuaaaaggcagauucuAa........................................................    1        1     seq
    .......................cuuguuaaaaggcagauucu..........................................................    1        0     seq
    .......................................uucugauucagggaccaagauuc.......................................    1        0     seq
    ........................................ucugauucagggaccaagauucu......................................    1        0     seq
    .........................................cugauucagggaccaagauucugc....................................    1        0     seq
    ..........................................................auucugcauuuuuagcaaguu......................    3        0     seq
    ..........................................................auucugcauuuuuagcaaguuU.....................    2        1     seq
    ..........................................................auucugcauuuuuagcaaguuc.....................    13       0     seq
```
